# Morin Flavonoid Interaction with Albumin and Its Nanoparticle Conjugation: An Efficient Antioxidant Vehicle for Nutraceuticals

**DOI:** 10.3390/antiox14070764

**Published:** 2025-06-21

**Authors:** Guillermo Montero, Víctor Guarnizo-Herrero, Catalina Sandoval-Altamirano, Germán Günther, Soledad Bollo, Francisco Arriagada, Javier Morales

**Affiliations:** 1 Facultad de Ciencias, Universidad San Sebastián, Santiago 7510157, Chile; guillermo.montero@uss.cl; 2 Facultad de Farmacia, Universidad de Alcalá, 28805 Alcalá de Henares, Spain; victor.guarnizo@uah.es; 3 Facultad de Química y Biología, Universidad de Santiago de Chile, Santiago 9170002, Chile; catalina.sandoval.a@usach.cl; 4 Facultad de Ciencias Químicas y Farmacéuticas, Universidad de Chile, Santiago 8380492, Chile; ggunther@ciq.uchile.cl (G.G.); sbollo@ciq.uchile.cl (S.B.)

**Keywords:** morin, flavonoid, albumin nanoparticles, nutraceuticals, encapsulation, functional food

## Abstract

Morin is a natural flavonoid with potent antioxidant activity, yet its clinical and nutraceutical applications remain limited due to poor aqueous solubility and low bioavailability. This study explores the interaction of morin with bovine serum albumin (BSA) and the development of BSA-based nanoparticles as a delivery platform. Fluorescence spectroscopy confirmed the formation of a stable 1:1 morin–BSA complex, governed by hydrophobic interactions, with a binding constant (Ka) of 1.87 × 10^5^ L·mol^−1^. Binding conferred enhanced photostability, as BSA attenuated morin degradation under oxidative stress conditions. BSA nanoparticles prepared by desolvation encapsulated morin with high monodispersity and encapsulation efficiencies up to 26%. Co-encapsulation with ellagic acid or tocopherol succinate improved loading capacity but reduced morin release, suggesting intermolecular stabilization. Release studies in simulated intestinal fluid showed controlled diffusion, while compatibility assays in milk-based food matrices confirmed colloidal stability in whole and reduced-fat milk. These findings support BSA–morin nanoparticles as a promising system for the oral delivery and functional food incorporation of polyphenolic antioxidants.

## 1. Introduction

**Figure 1:**
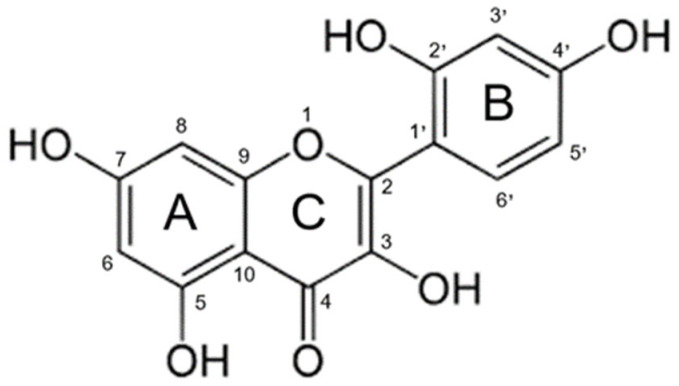
Chemical structure of morin.

Morin (3,5,7,2′,4′-pentahydroxyflavone) (Figure 1) is a naturally occurring flavonol found in fruits, vegetables, tea, wine, and various medicinal herbs [1]. It has been isolated from plant species such as *Maclura pomifera*, *Maclura tinctoria*, and *Psidium guajava*, and has attracted increasing interest in the biomedical and pharmaceutical fields due to its broad spectrum of bioactive properties [2]. However, despite its promising therapeutic profile, its clinical application has been significantly limited by its low oral bioavailability [3]. This deficiency (weakness) is attributed to its poor aqueous solubility, chemical instability under adverse environmental conditions, and limited intestinal permeability [4].

Morin has demonstrated a wide range of pharmacological activities, including antioxidant, anti-inflammatory, antiallergic, antiviral, and anticancer properties [5,6]. These effects are particularly relevant in the context of chronic and inflammatory diseases, where oxidative stress plays a central role [7]. Nonetheless, the therapeutic exploitation of these benefits largely depends on the structural stability of morin and its appropriate biodistribution in the body.

An important strategy to improve the bioavailability of flavonoids, such as morin, involves studying their interaction with plasma proteins, which modulate the transport, distribution, and metabolism of drugs and bioactive compounds. These interactions can alter both the chemical stability and the biological activity of flavonoids. Several studies have shown that flavonoids and polyphenols can bind to proteins such as caseins, digestive enzymes, and notably albumin, generating synergistic or modifying effects on their pharmacokinetics and redox behavior.

In this context, serum albumin—the most abundant protein in human and animal plasma—represents a natural platform with high binding capacity for a wide variety of endogenous and exogenous compounds. Bovine serum albumin (BSA) has been extensively used as an experimental model to study drug–protein interactions due to its high structural homology (76%) with human serum albumin (HSA). BSA is a globular protein composed of three homologous domains, each subdivided into subdomains A and B, which contain multiple ligand-binding sites. This protein has two tryptophan residues (Trp134 and Trp212), responsible for its intrinsic fluorescence, making it an ideal system for characterizing molecular interactions using fluorescence spectroscopy [8,9,10].

The physicochemical properties of albumin, such as solubility in aqueous media, thermal stability (up to 60 °C), and structural resilience over a wide pH range (4–9), position it as an excellent matrix for the incorporation and transportation of bioactive compounds. Furthermore, its biodegradability and biocompatibility make it suitable for both pharmaceutical and nutraceutical applications [11].

The interaction between flavonoids and albumin not only affects compound distribution but also influences their stability against oxidative processes. It has been shown that such association can protect polyphenols from oxidation induced by reactive oxygen species (ROS), although, in some cases, it may increase susceptibility by exposing functional groups that are sensitive to degradation [12]. Structural features of flavonoids, such as hydroxyl or methoxy groups, determine their binding affinity to albumin and their ability to displace other ligands, potentially inducing conformational changes in the protein [12].

Polyphenols are particularly vulnerable to oxidative degradation, a process that diminishes their efficacy and may lead to the formation of toxic products. In the case of morin, exposure to light, alkaline conditions, or elevated temperatures can result in isomerization, cyclization, or peroxide formation. Protection against such degradation processes is essential to preserve its functionality as an antioxidant agent. Various strategies have been proposed to mitigate this phenomenon, including the use of proteins such as albumin as protective barriers, and more recently, the development of nanostructured delivery systems [13].

Nanotechnology has created new opportunities for formulating unstable bioactive compounds by producing nanoparticles that enhance their solubility, protecting them from harsh environmental conditions, and enabling controlled release. Among these platforms, albumin-based nanoparticles have shown promising results due to their ability to encapsulate both hydrophobic and hydrophilic compounds while preserving their integrity and functionality. The simple coacervation technique has been widely employed to produce BSA nanoparticles due to its straightforward procedure, precise size control, and high reproducibility [14,15].

In the case of natural antioxidants, albumin nanoparticles not only enable efficient encapsulation but also enhance their therapeutic properties through structural stabilization [13]. This is particularly relevant in the development of nutraceutical products, where preserving the biological activity of compounds during storage, transport, and administration is essential. Moreover, the versatility of these nanoparticles enables their formulation in various formats, including colloidal suspensions and lyophilized powders, making them suitable for multiple administration routes [16].

In this context, the present study aimed to characterize the interaction between morin and bovine serum albumin (BSA) using fluorescence spectroscopy and surface plasmon resonance (SPR), and to evaluate the effect of this interaction on the photooxidative stability of morin. This work seeks to provide key insights into the rational design of albumin-based nanosystems incorporating morin, with potential applications in developing stable and effective nutraceutical formulations.

## 2. Materials and Methods

Morin (MOR, ≥99%) and bovine serum albumin (BSA, fraction V, ≥98%) were obtained from Merck (Darmstadt, Germany). The complementary antioxidant compounds (quercetin, kaempferol, genistein, genistin, coumestrol, ellagic acid, and tocopherol succinate) used in this work were obtained from Sigma-Aldrich (St. Louis, MO, USA). HPLC-grade solvents such as methanol, ethanol, and acetonitrile were purchased from Merck (Darmstadt, Germany). Dimethyl sulfoxide (DMSO) and trifluoroacetic acid (99%) from Sigma-Aldrich (Darmstadt, Germany) and hydrogen peroxide (30% solution) from Merck (Billerica, MA, USA) were reagent grade. Deionized water (18.2 MΩ cm) was used throughout. All reagents were used without further purification.

### 2.1. Fluorescence Spectroscopy and Thermodynamic Analysis of Albumin–Morin Interactions

Fluorescence quenching spectroscopy was employed to investigate the interaction between bovine serum albumin (BSA) and selected flavonoids, including morin. All measurements were performed using a spectrofluorometer under controlled temperature conditions (25 °C). The intrinsic fluorescence of BSA was monitored by excitation at 295 nm to selectively detect tryptophan residues, and the emission spectra were recorded in the range of 300–450 nm [17,18].

To quantify the quenching effect upon encapsulation, the Stern–Volmer equation was applied (Equation (1)):

F_0_/F = 1 + Ksv [Q] (1)

where F_0_ and F are the fluorescence intensities of BSA in the absence and presence of quencher (flavonoid), respectively, Ksv is the Stern–Volmer quenching constant, and [Q] is flavonoid concentration.

The binding constant (Ka) and the number of binding sites (n) were calculated using the logarithmic form of the binding equation (Equation (2)):

log[(F_0_ − F)/F] = log Ka + n log[Q] (2)

Thermodynamic parameters—Gibbs free energy change (ΔG°) (Equation (3)), enthalpy change (ΔH°), and entropy change (ΔS°)—were determined from the temperature dependence of the binding constant using the van’t Hoff equation (Equation (4)):

ΔG° = ΔH° − TΔS°(3)

ln Ka = −ΔH°/RT + ΔS°/R(4)

These parameters provided insight into the intermolecular forces governing the interaction, including hydrophobic, electrostatic, and hydrogen bonding contributions.

### 2.2. Surface Plasmon Resonance (SPR) Analysis of Albumin–Morin Interactions

Surface plasmon resonance (SPR) experiments were carried out using a dual-channel SPR 7500DC instrument (Reichert Technologies, Depew, NY, USA) to investigate the binding interaction between bovine serum albumin (BSA) and morin. Gold-coated sensor chips were first cleaned and subsequently modified with a self-assembled monolayer (SAM) of 4-mercaptobenzoic acid (4-MBA) to introduce carboxyl functional groups on the sensor surface.

Covalent immobilization of BSA onto the functionalized surface was achieved using standard carbodiimide coupling chemistry. Briefly, the carboxylic acid groups were activated with a 1:1 mixture of N-(3-dimethylaminopropyl)-N′-ethylcarbodiimide hydrochloride (EDC) and N-hydroxysuccinimide (NHS), followed by the injection of BSA (0.1 mg/mL in acetate buffer, pH 5.0). Non-reacted sites were blocked with 1 M ethanolamine (pH 8.5).

Interaction analyses were performed by injecting morin solutions at increasing concentrations, ranging from 50 to 500 µM, prepared in phosphate-buffered saline (PBS, pH 7.4) containing 5% (*v*/*v*) dimethyl sulfoxide (DMSO) to improve compound solubility. Sensorgrams were recorded in real time, and binding responses were referenced against control channels and buffer-only injections.

Equilibrium binding data were analyzed using Langmuir isotherm models to estimate the apparent association constant (Ka), dissociation constant (Kd), and maximum binding response (Rmax). Data fitting and kinetic analysis were performed using the instrument’s integrated software suite [19,20,21].

### 2.3. Photolysis and Scavenging of Hydroxyl Radical by Ultraviolet Radiation

To investigate the time-dependent degradation of morin under UV-induced photolysis and its reactivity with hydroxyl radicals, aqueous solutions of morin were prepared in 100 mM phosphate buffer (pH 7.4), either with or without bovine serum albumin (BSA). Morin was incorporated into the system by adding small aliquots of a stock solution in ethanol, ensuring the final ethanol concentration remained below 2% (*v*/*v*) to avoid solvent-induced effects on photoreactivity. Oxidative conditions were simulated by the addition of hydrogen peroxide to a final concentration of 0.5% (*w*/*v*), serving as a source of hydroxyl radicals upon UV activation. Each reaction mixture was vortexed to ensure homogeneity and subsequently transferred into 5 mL double-jacketed quartz cuvettes. The outer surface of each cuvette was coated with opaque black paint to prevent ambient light interference, except for a centrally located circular window (1 cm diameter) designed to permit focused irradiation. A circulating water bath maintained the cuvette temperature at 20 ± 0.5 °C. Photolysis was carried out using a high-power mercury UV lamp emitting at 254 nm. Samples were irradiated for predetermined time intervals, and aliquots were collected at each time point, immediately protected from light, and analyzed. Quantitative analysis of morin degradation and spectral changes associated with hydroxyl radical attack was performed using high-performance liquid chromatography with diode array detection (HPLC-DAD) and UV–visible spectrophotometry.

### 2.4. Circular Dichroism (CD) Spectroscopy

Circular dichroism (CD) spectra in the far-UV region (190–260 nm) were recorded to assess potential conformational changes in bovine serum albumin (BSA) induced by its interaction with morin and oxidative stress. Measurements were performed using a J-1500 CD spectropolarimeter (JASCO, Tokyo, Japan) equipped with a temperature-controlled cell holder maintained at 25 °C. Spectra were acquired using a 0.1 cm path length quartz cuvette. Each spectrum represents the average of three consecutive scans, recorded at a scanning speed of 100 nm/s with a response time of 1 s. The final BSA concentration was fixed at 2 μM, and samples were prepared at different BSA-to-morin molar ratios: 1:0 (control), 1:5, and 1:10. For oxidative conditions, hydrogen peroxide was added to a final concentration of 0.5% (*w*/*v*) to promote hydroxyl radical generation. All solutions were prepared in 10 mM phosphate buffer at pH 7.4 and allowed to equilibrate for 15 min before measurement. Spectra were corrected by subtracting the baseline of the corresponding buffer or hydrogen peroxide control solution. CD data were analyzed to assess secondary structural alterations of BSA in the presence of morin and under oxidative conditions [22,23,24].

### 2.5. Morin Analysis

A high-performance liquid chromatography (HPLC) method was developed and validated to quantify morin for the subsequent evaluation of encapsulation efficiency, drug loading capacity, physicochemical stability, photodegradation under ultraviolet (UV) exposure, and hydroxyl radical scavenging activity. Chromatographic analyses were conducted using a Shimadzu LC-20AT HPLC system (Shimadzu, Kyoto, Japan), equipped with a SIL-20A autosampler and an SPD-M20A photodiode array (PDA) detector. Separation was achieved on a C18 ODS Hypersil column (250 mm × 4.6 mm, 5 μm particle size; Agilent Technologies, Santa Clara, CA, USA). The mobile phase consisted of acetonitrile and 0.1% trifluoroacetic acid in water (30:70, *v*/*v*), delivered under isocratic conditions at a flow rate of 1.0 mL/min. The injection volume was set at 20 μL. Morin detection was performed at a wavelength of 360 nm using the PDA detector. The method was optimized to ensure adequate peak resolution, retention time reproducibility, and sensitivity for low-concentration samples. This analytical approach was employed throughout the study to monitor morin concentration in various experimental settings, including nanoparticle formulations and stability assays.

### 2.6. In Vitro Antioxidant Behavior of Morin in the Presence of Other Antioxidant Compounds

The impact of other antioxidant molecules (ellagic acid and tocopherol succinate) on the behavior of morin encapsulated in BSA nanoparticles in an intestinal environment was assessed through in vitro loading and release experiments. First, 1.25 mg of morin was mixed with a 25 mg/mL BSA solution at pH 9.0 for 30 min at room temperature. Then, ethanol was added dropwise under constant stirring, followed by the addition of 20 µL of an 8% glutaraldehyde solution [25]. The final volume of the mixture was adjusted to 10 mL. After 24 h, the nanoparticles were collected by centrifugation (13,500 rpm, 20 min, and 25 °C) and washed with water. The supernatant was separated for further analysis. To incorporate ellagic acid or tocopherol succinate, each compound—either individually or in combination—was included in the ethanol phase during the dropwise addition. The rest of the procedure remained unchanged. The encapsulation efficiency (%EE) was calculated using Equation (5):

(5) $$\%EE=\frac{M_{i}-M_{f}}{M_{i}}\times 100$$

where M_i_ and M_f_ correspond to the initial amount of morin added during the loading process and the free morin in the supernatant, respectively.

Second, the release of morin in a simulated intestinal fluid (SIF) without enzymes, as established by the USP, was evaluated using a sample-and-separation method. Morin-loaded nanoparticles and different combinations with ellagic acid and/or tocopherol succinate were suspended in 20 mL of release medium at 37 °C under constant stirring at 100 rpm. At predefined time points (0.5, 1, 2, 3, 4, 5, and 6 h), 2 mL samples were withdrawn and centrifuged. The supernatant was separated, and the pellet was resuspended in 2 mL of fresh release medium at the same temperature and returned to the release assay. The analysis was performed considering the mean ± standard deviation (SD) of at least three independent experiments. All supernatants obtained during the loading or release process were quantified using the HPLC method described above.

### 2.7. Characterization of Nanoparticles

The hydrodynamic diameter, polydispersity index (PDI), and zeta potential of the nanoparticles (NPs) were determined by dynamic light scattering (DLS) using a Zetasizer Nano ZS90 instrument (Malvern Instruments, Malvern, UK). Measurements were performed at 25 °C following appropriate dilution with ultrapure water to avoid multiple scattering effects. Each sample was analyzed in triplicate, and results were reported as mean ± standard deviation.

The surface morphology and structural features of the nanoparticles were examined by scanning electron microscopy (SEM) using a FEI Inspect F50 microscope. Prior to analysis, samples were deposited onto aluminum stubs, air-dried, and coated with a thin layer of gold under vacuum using a sputter coater to enhance conductivity. Representative micrographs were acquired at various magnifications to assess particle shape, aggregation, and surface texture.

### 2.8. Statistical Analysis

Data are presented as the mean ± standard deviation (SD) of at least three independent measurements.

## 3. Results and Discussion

### 3.1. Morin–Albumin Interaction: Fluorescence and Surface Plasmon Resonance Studies

Fluorescence and SPR experiments were used to assess the interaction between BSA and a series of structurally related flavonoids: morin, quercetin, kaempferol, genistein, and genistin. These flavonoids differ in their hydroxylation patterns and glycosylation, factors that are known to influence protein-binding affinity.

**Figure 2:**
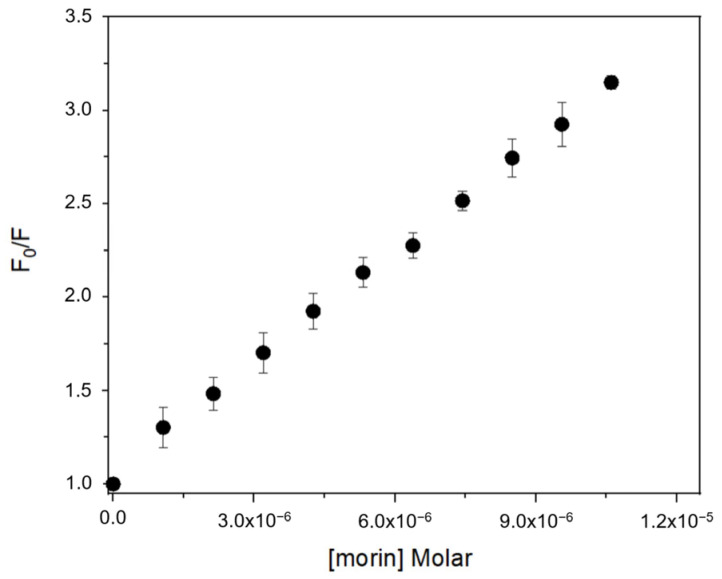
Fluorescence quenching Stern–Volmer plot of BSA with increasing concentration of morin at 25 °C.

Fluorescence spectra of BSA exhibited a progressive decrease in emission intensity upon titration with each flavonoid, consistent with complex formation [18,26,27]. The quenching data fitted well to the Stern–Volmer equation (Table 1), confirming a static quenching mechanism in all cases. Calculated Stern–Volmer constants (K_SV_) and apparent association constants (Ka) showed that flavonols (morin, quercetin, kaempferol) bind with higher affinity than isoflavones (genistein, genistin) (Figure 2). Morin exhibited the highest Ka value (1.87 × 10^5^ L·mol^−1^), followed by quercetin (1.73 × 10^5^ L·mol^−1^) and kaempferol (1.67 × 10^5^ L·mol^−1^), while genistein and genistin displayed lower Ka values (8.78 × 10⁴ and 3.39 × 10⁴ L·mol^−1^, respectively). This trend suggests that the position of the B ring and the presence of free hydroxyl groups at positions 3′ or 2′ are key determinants of binding affinity.

Thermodynamic parameters were estimated for each compound based on van’t Hoff analysis at multiple temperatures [22,23]. All binding processes were spontaneous (ΔG° < 0) and mainly driven by hydrophobic forces, as evidenced by negative ΔH° and positive ΔS° values [28,29,30]. For instance, the morin–BSA complex had ΔH° = –28.7 kJ·mol^−1^ and ΔS° = 4.75 J·mol^−1^·K^−1^, resulting in ΔG° values of approximately –30.1 kJ·mol^−1^ across temperatures from 293–310 K. Comparable values were obtained for quercetin and kaempferol. The reduced ΔG° magnitude for genistin (–26.9 kJ·mol^−1^) supports the hypothesis that glycosylation impairs protein affinity [26,31].

On the other hand, SPR experiments further confirmed these findings [19,32]. Morin was the only compound among those tested that reached measurable equilibrium binding under the experimental conditions, due to its higher aqueous solubility at pH 7.4 [33]. A Langmuir model yielded a Ka of 3.51 × 10^3^ L·mol^−1^ for morin–BSA, which is two orders of magnitude lower than the value obtained via fluorescence. This discrepancy is attributed to the limited accessibility of BSA binding sites when immobilized on the SPR sensor chip, in contrast to the freely diffusing BSA in solution. Similar phenomena have been reported for ellagic acid and its metabolites using SPR, where Ka values ranged between 3 × 10^3^ and 6 × 10^3^ L·mol^−1^ [19]. Shamsi et al. demonstrated, through fluorescence studies, a high binding affinity between human serum albumin (HSA) and rosmarinic acid (RA), with an association constant (K) on the order of 10^7^ M^−1^. The thermodynamic parameters obtained indicated that hydrophobic interactions play a predominant role in this binding process [34]. In contrast, Hornok et al. evaluated the interaction of kynurenic acid (KYNA) with HSA from both structural and thermodynamic perspectives, employing fluorescence spectroscopy, surface plasmon resonance (SPR), and circular dichroism. Their results revealed the formation of a low-affinity complex with two independent binding sites and showed that the presence of the drug induces significant structural changes in the protein [35].

Summarizing, these results reveal that structural elements such as the number and position of hydroxyl groups, as well as glycosylation, significantly affect flavonoid–albumin binding. Moreover, methodological differences between solution-based and surface-based systems must be considered when interpreting affinity data. Fluorescence spectroscopy appears to be more sensitive for detecting high-affinity interactions, whereas SPR is limited by immobilization constraints and solubility issues but remains a valuable tool for monitoring real-time binding kinetics.

These insights are particularly relevant for the design of albumin-based delivery systems for flavonoids, especially those with limited oral bioavailability. Morin, due to its strong and thermodynamically favorable interaction profiles, emerges as a promising candidate for further development.

### 3.2. Morin Stability and Antioxidant Studies: Photolysis and Scavenging of Hydroxyl Radical by Ultraviolet Radiation

Polyphenols, particularly flavonoids such as morin, are highly susceptible to oxidative degradation, which can compromise their antioxidant capacity and therapeutic potential [36,37]. In biological systems, their interaction with plasma proteins like bovine serum albumin (BSA) may modulate their chemical stability and photodegradation behavior [12]. Previous studies on the reactivity of flavonoids as quenchers of singlet oxygen generated by photosensitization have shown morin as a highly effective natural antioxidant, exhibiting greater reactivity than quercetin. This enhanced activity is attributed to the electronic activation of the C2–C3 double bond in the C ring, promoted by electron delocalization from the hydroxyl group at R4′ in the B ring into the conjugated π system. This delocalization activates the ortho and para positions (C-3′, C-5′, and C-1′), increasing electron density at the C2–C3 bond. Additionally, the hydroxyl group at R3 in the C ring further contributes, enhancing the nucleophilicity of the system and its antioxidant performance [33].

**Figure 3:**
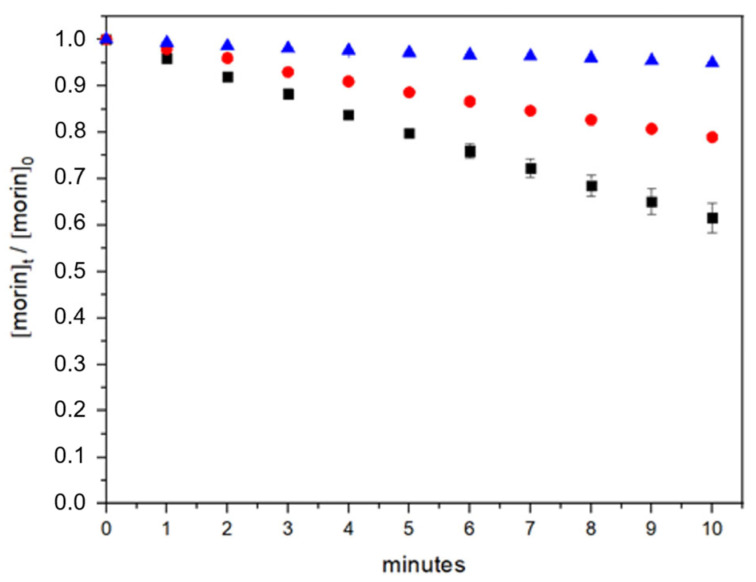
Morin degradation kinetics over time under different conditions: direct photolysis (blue), photooxidation in the absence of BSA (black), and photooxidation in the presence of BSA (red).

In this work, to assess the oxidative stability of morin and the potential protective effect conferred by bovine serum albumin (BSA), we evaluated its degradation kinetics (Equation (6)) under photolysis and photooxidation (reactivity against hydroxyl radicals generated by hydrogen peroxide photolysis) conditions in phosphate buffer at pH 7.4 (Figure 3) [38]. Experiments were performed both in the absence and presence of BSA to determine whether protein binding influences morin stability.

(6) $$v=\;-\frac{d\;\left[morin\right]}{dt}=\;k\;\times \left[{HO}^{•}\right]\left[morin\right]=\;k_{obs}\left[morin\right]$$

In the absence of BSA, morin demonstrated substantial stability under UV photolysis. After 10 min of constant light exposure, 94.9% of the initial morin concentration remained, indicating that photolytic degradation alone is not a major pathway for compound loss. In contrast, the presence of hydrogen peroxide during UV exposure dramatically increased the degradation rate, reducing the morin concentration to 61.5% after the same period. These results confirm that oxidative degradation is the primary mechanism responsible for morin breakdown under these conditions.

The incorporation of BSA significantly mitigated the extent of photooxidation. In the presence of 2 µM BSA, 79% of the initial morin concentration was retained after 10 min of UV exposure with hydrogen peroxide, suggesting a protective role. This corresponds to a 17.5% improvement in morin retention compared to the system without BSA. Such stabilization may be attributed to non-covalent interactions between morin and the protein, limiting the access of reactive oxygen species (ROS) to the antioxidant molecule.

This protective effect is consistent with previous studies that describe albumin as a carrier and stabilizer of bioactive compounds. Arriagada et al. proposed that morin acts via a proton-coupled electron transfer (PCET) mechanism, providing radical scavenging capacity under oxidative stress [39,40]. The current findings support the notion that this antioxidant mechanism is preserved and possibly enhanced by the interaction with BSA.

Interestingly, not all studies report a protective effect. Yu et al. found that BSA-stabilized platinum nanoparticles actually promoted morin oxidation, attributing this to increased surface interactions and enhanced ROS generation at the nanoparticle interface [41]. This highlights the significance of system-specific behavior, where albumin’s role can vary depending on the physicochemical environment and the presence of catalytic surfaces.

**Figure 4:**
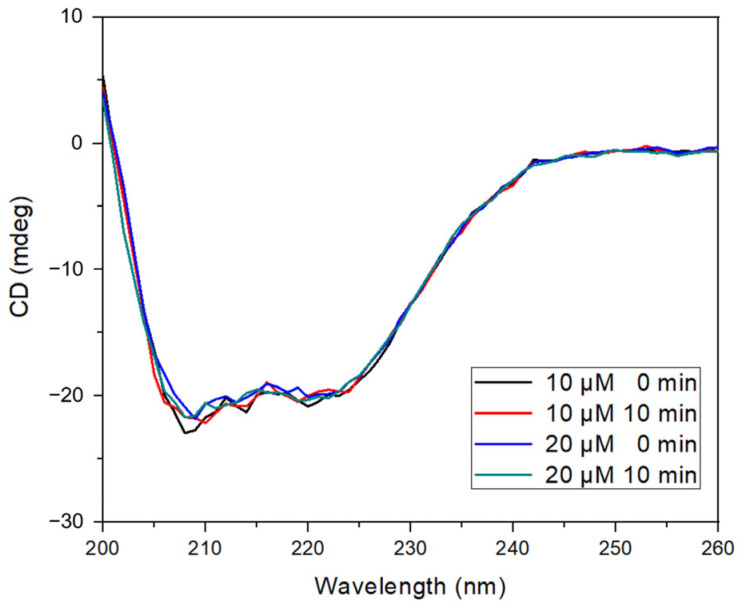
BSA–morin CD spectra. Reaction with hydroxyl radical generated by photolysis of H_2_O_2_ at fixed BSA concentration and varying morin concentrations at 0 and 10 min.

To further analyze the interaction, circular dichroism (CD) spectroscopy was used to assess the impact of morin binding on the secondary structure of BSA (Figure 4). The characteristic negative ellipticity bands observed at 208 and 222 nm—attributed to α-helical content—were preserved in all experimental conditions, including UV irradiation and the presence of hydrogen peroxide. The addition of morin (10 and 20 µM) resulted in a slight reduction in ellipticity intensity without shifting the spectral maxima, indicating no significant loss of α-helical structure or induction of protein unfolding [24,42].

These results suggest that the morin binding does not cause conformational destabilization, even under oxidative conditions, and that the protein maintains its structural integrity while stabilizing morin.

In summary, these findings confirm that BSA can attenuate the oxidative degradation of morin, supporting its role as a stabilizing excipient in pharmaceutical formulations containing sensitive polyphenolic compounds.

### 3.3. Morin–Albumin Nanoparticles: Preparation, Characterization, and Release

Bovine serum albumin (BSA) nanoparticles loaded with morin were successfully prepared using the desolvation technique [43]. The hydrodynamic size and zeta potential of the nanosystem could be tuned by modulating key process parameters, including BSA concentration, pH, ionic strength, degree of crosslinking (via glutaraldehyde concentration), and the type, concentration, and injection rate of the desolvating agent [44]. Ethanol was selected as the desolvating agent based on its well-documented use in the preparation of albumin nanoparticles. BSA nanoparticles (without morin) were synthesized under the same conditions and served as experimental controls.

**Figure 5:**
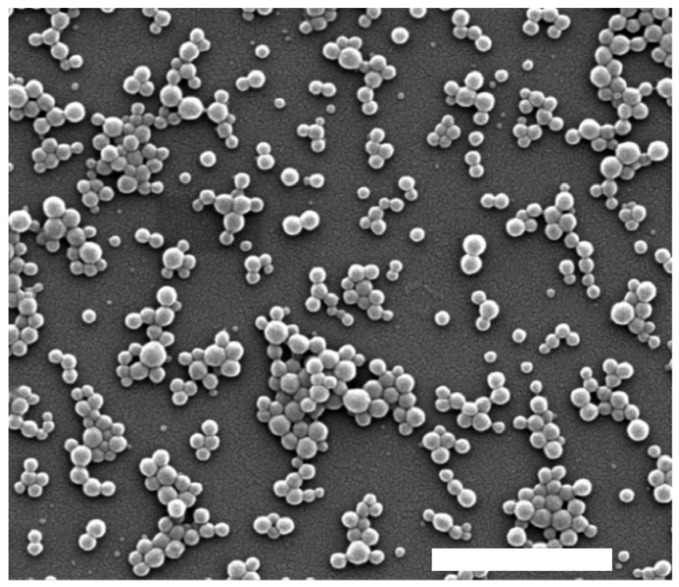
SEM micrograph of albumin–morin nanoparticles. Scale bar: 1 µm.

Table 2 shows that all synthesized nanoparticles exhibited a low polydispersity index (PdI), indicative of a high degree of monodispersity and uniform size distribution. Specifically, nanoparticles containing morin (morin-BSA NPs) exhibited an average hydrodynamic diameter of 118.1 nm with a narrow size distribution. This size was greater than that observed for BSA nanoparticles and than that in previous studies with coumestrol-loaded nanoparticles [42]. A similar trend was observed for zeta potential values, which showed lower absolute values for morin-BSA NPs compared to the other systems. Zeta potential measurements were negative for loaded and unloaded NPs, with less negative values observed in the absence of morin. This negative surface charge is attributed to ionized functional groups on the BSA surface, which contribute to colloidal stability by preventing aggregation. SEM imaging confirmed the spherical morphology of morin-BSA NPs (Figure 5).

The encapsulation efficiency (%EE) of morin was 20.99% ± 0.27% for an initial concentration range of 0.1 to 1.5 mM morin, added to a fixed amount of BSA (Table 3). Based on the loading efficiency of the nanosystem, it was observed that increasing the concentration of incorporated morin leads to a molar ratio of morin to BSA (morin/BSA) of 1:1 at concentrations above 1 mM. This result is consistent with the interaction studies conducted using fluorescence spectroscopy, which indicate the presence of a single binding site per morin molecule on the protein. These findings confirm a 1:1 interaction stoichiometry between serum albumin and the flavonoid morin.

Encapsulation efficiency is influenced by multiple parameters, including the chemical nature of the bioactive compound, the molecular characteristics of the nanoparticle matrix, the solvent or solvent mixtures used, and the loading technique applied. Previous studies reported that the encapsulation efficiency of the phytoestrogen coumestrol in BSA nanoparticles was 22.4% ± 0.1%, equivalent to approximately three coumestrol molecules per ten BSA molecules [42]. For instance, Ghosh et al. reported the incorporation of fisetin—a flavonoid with antioxidant properties—into BSA nanoparticles prepared via desolvation, achieving an average particle size of 200 ± 8 nm and an encapsulation efficiency of 84% [45].

To compare with previous systems, BSA nanoparticles co-loaded with morin and coumestrol were prepared under the same experimental conditions used for individual compound encapsulation. Both compounds were introduced simultaneously into the formulation, and the %EE for each was subsequently determined by independent HPLC methods specific for each compound. Coumestrol exhibited an encapsulation efficiency of 22.4% ± 0.1%, consistent with previously reported values, and remained unchanged when co-loaded with morin. Similarly, the %EE of morin did not vary significantly when encapsulated alone (20.5% ± 3.0%) or in the combined formulation. These findings suggest the absence of competitive interactions between the two flavonoids for binding sites within the BSA matrix, enabling their simultaneous incorporation into a single nanocarrier system. To further enhance the encapsulation efficiency (%EE) of morin in BSA nanoparticles, optimization of the desolvation process could be explored. Specifically, adjusting the rate of solvent addition or extending the drug loading time [44,46]. These strategies warrant further investigation to balance encapsulation efficiency with nanoparticle stability and scalability. To assess the robustness of morin encapsulation efficiency and its release from albumin nanoparticles, a competitive binding evaluation was performed using two additional antioxidants: ellagic acid and tocopherol succinate. These compounds were selected due to their potential to interfere with BSA–morin interactions based on their distinct structural characteristics. Ellagic acid is a polar polyphenolic compound bearing four hydroxyl groups, while tocopheryl succinate consists of a hydrophobic hydrocarbon chain attached to a chromanol ring.

**Figure 6:**
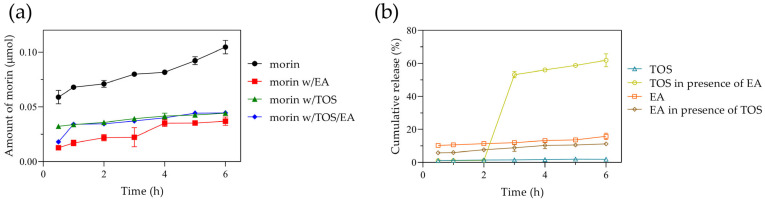
(**a**) Amounts of morin diffused from nanoparticles. Morin loaded alone (circle), morin and ellagic acid co-loaded (square), morin and tocopherol succinate co-loaded (triangle), and morin and ellagic acid/tocopherol succinate co-loaded (diamond). (**b**) Cumulative release of tocopherol succinate and ellagic acid from BSA nanoparticles. Tocopherol succinate co-loaded with morin (open triangle), tocopherol succinate co-loaded with morin and ellagic acid (open circle), ellagic acid co-loaded with morin (open square), and ellagic acid co-loaded with morin and tocopherol succinate (open diamond).

A potential application of this nanosystem presented is the transport of antioxidants through the gastrointestinal tract; therefore, we evaluated the behavior of morin in a simulated intestinal medium over a typical intestinal transit time of 6 h. Approximately 21% of morin was released within the first 30 min, increasing to 25% at 3 h and reaching 30% at 6 h. These results are comparable to those reported by Ghosh P. et al. at pH 7.4 [13]. Despite differences in the release media, the initial 6 h release in both studies remained low (<40%), with an extended release of up to 80% at 72 h at pH 7.4. In our study, the amount of morin released from the albumin nanoparticles ranged from 0.063 to 0.1 µmol (Figure 6a), following zero-order kinetics.

When ellagic acid was incorporated, the %EE of morin increased to 76%, likely due to interactions involving -OH groups or π-π stacking interactions. Similarly, tocopherol succinate incorporation resulted in a morin %EE of 72.6%, while the simultaneous presence of both ellagic acid and tocopherol succinate led to a morin %EE of 80%. However, the amount of morin released from the nanoparticles decreased significantly, reaching only ~0.03 µmol, indicating that morin alone was released three times more efficiently. Overall, both encapsulated ellagic acid and tocopherol succinate exhibited low release, with 12% and 2%, respectively. Interestingly, when all three compounds were co-encapsulated, both ellagic acid and morin demonstrated a slow and steady release, whereas tocopherol succinate exhibited a latency phase, with a burst release of ~55% at 3 h, reaching 60% at 6 h (Figure 6b).

The results indicate that morin encapsulation improves when components capable of complex formation enhance interactions between themselves and the protein rather than with the solvent. In this regard, Ghaeh F.S. et al. reported high tocopherol encapsulation in BSA nanoparticles [47], while Liu F. et al. described strong interactions between ellagic acid and BSA [48], which promoted high encapsulation efficiency of ellagic acid or its complexes, as observed in our study with morin. Similarly, Oliveira et al. leveraged the lipophilic characteristics of alpha-tocopherol to enhance doxorubicin encapsulation [49]. While morin incorporation improved and the encapsulation of ellagic acid and tocopherol was high, their diffusion from the nanosystem was restricted. Consistent with our findings, previous studies have shown that due to their hydrophobic nature, such molecules exhibit slow release from polymeric or protein-based particles during the initial hours [50,51,52]. In this regard, the release behavior can be controlled by the system’s degradation within the gastrointestinal tract. Overall, the results demonstrate that the nanoparticles efficiently load, transport, and retain morin alongside other compounds in a simulated intestinal environment, exhibiting minimal diffusion from the nanosystem. This highlights the importance of nanomaterial engineering modifications to regulate the delivery of bioactive molecules according to specific needs. Further studies on release behavior in different media or strategies to enhance release, if necessary, are beyond the scope of this work.

### 3.4. Incorporation of BSA–Morin Nanoparticles into Food Matrices Based on Dispersed Systems

Different concentrations of the BSA–morin nanosystem were incorporated into food matrices composed of liquid–liquid dispersed systems (milk with varying fat contents) to evaluate the compatibility and colloidal stability of the system after nanoparticle addition. The nanoparticles were added at five concentration levels ranging from 0.2 to 2% *w*/*w*. Stability was monitored through visual inspection, microscopy, viscosity, and analysis of potential interactions with native components of the food matrix.

The results demonstrate that bovine serum albumin nanoparticles loaded with morin (BSA–morin NPs) are compatible with whole and reduced-fat milk matrices (Table 4). In these systems, no signs of colloidal instability or visible alterations were observed, indicating successful incorporation of the nanosystem without compromising the integrity of the food matrix. In contrast, the appearance of a precipitate in skim milk following nanoparticle addition suggests an unfavorable interaction, likely due to the lower lipid content, which may impair nanoparticle dispersion or stabilization.

A slight increase in viscosity was observed upon nanoparticle incorporation, which may be attributed to physical interactions between the BSA–morin NPs and lipid or protein components of the matrix. Furthermore, a faint shift in color toward a creamy-yellow hue was consistently noted in all nanoparticle-treated samples, aligning with the intrinsic pigmentation of morin, a naturally occurring flavonoid.

Overall, these findings support the potential application of BSA–morin NPs in the formulation of functional foods, provided that the composition of the food matrix—particularly its fat content and emulsion viscosity—is taken into account. The observed modulation of the physicochemical behavior of the nanosystem by the food matrix highlights the importance of tailoring formulation strategies to the specific characteristics of the target food product.

## 4. Conclusions

This study demonstrates that morin exhibits a high binding affinity to bovine serum albumin (BSA), primarily through hydrophobic interactions, as evidenced by fluorescence spectroscopy, surface plasmon resonance (SPR), and thermodynamic analyses. The formation of a stable 1:1 morin–BSA complex contributes to the improved photooxidative stability of morin in aqueous environments, suggesting a protective role of albumin against reactive oxygen species.

BSA-based nanoparticles prepared by the desolvation method efficiently encapsulated morin, achieving favorable physicochemical properties, including monodisperse size distribution, negative surface charge, and spherical morphology. The encapsulation efficiency and release kinetics of morin were modulated by co-loading with other antioxidant compounds such as ellagic acid and tocopherol succinate, without evidence of competitive binding. These results underscore the versatility of BSA nanoparticles for simultaneous delivery of multiple polyphenols with distinct physicochemical profiles.

Morin-loaded BSA nanoparticles demonstrated structural stability and low diffusion rates in simulated intestinal media, supporting their suitability for oral delivery applications.

Furthermore, their compatibility with dairy matrices—particularly milk with moderate to high fat content—underscores their feasibility for integration into functional food systems. However, despite these promising in vitro results, further in vivo studies are essential to validate the bioavailability, pharmacokinetics, and therapeutic potential of these nanoparticles when incorporated into nutraceutical products. Additionally, addressing scalability and cost-related challenges associated with the use of BSA—especially for food-grade or nutraceutical applications—is critical for future implementation. Altogether, this work establishes BSA nanoparticles as a robust and biocompatible platform that offers dual functionality: stabilization and sustained release of morin, along with compatibility with real-world food matrices. These findings pave the way for the development of next-generation nutraceutical formulations and functional foods enriched with flavonoids.

## Figures and Tables

**Table 1:** BSA-flavonoid binding parameters obtained from the Stern–Volmer equation (Stern–Volmer quenching constant (K_sv_), bimolecular quenching rate constant (k_q_), binding constant (Ka), and number of binding sites (n)).

Flavonoid	K_sv_ (L mol^−1^)	k_q_ (L mol^−1^ s^−1^)	K_a_ (L mol^−1^)	n
morin	1.76 × 10^5^ ± 0.03	1.76 × 10^13^ ± 0.02	1.87 × 10^5^ ± 0.02	1.10 ± 0.02
quercetin	1.88 × 10^5^ ± 0.02	1.88 × 10^13^ ±0.03	1.73 × 10^5^ ± 0.03	1.13 ± 0.02
kaempferol	2.23 × 10^5^ ± 0.03	2.23 × 10^13^ ± 0.02	1.67 × 10^5^ ± 0.02	1.33 ± 0.03
genistein	8.11 × 10^4^ ± 0.02	8.11 × 10^12^ ± 0.04	8.78 × 10^4^ ± 0.02	1.22 ± 0.04
genistin	3.66 × 10^4^ ± 0.05	3.66 × 10^12^ ± 0.04	3.39 × 10^4^ ± 0.01	0.94 ± 0.01

**Table 2:** Particle size, polydispersity index, zeta potential, and encapsulation efficiency of nanoparticles.

Nanoparticles	Diameter (Intensity) nm	Polydispersity Index (PdI)	ζ Potential (mV)	Encapsulation Efficiency
BSA	105.2 ± 23.2	0.092 ± 0.009	−47.6 ± 1.2	-
BSA-morin	118.1 ± 15.3	0.22 ± 0.04	−40.5 ± 1.4	20.99% ± 0.27%

**Table 3:** Effect of initial morin concentration on encapsulation efficiency (%EE) and morin/BSA molar ratio.

[Morin] (mM)	%EE	Morin/BSA
0.128	25.97 ± 4.29	0.30 ± 0.05
0.222	20.42 ± 0.67	0.48 ± 0.02
0.308	19.31 ± 0.42	0.68 ± 0.01
0.507	20.99 ± 0.27	0.98 ± 0.01
0.634	17.29 ± 0,63	1.01 ± 0.04
1.478	18.94 ± 0.85	1.48 ± 0.07

**Table 4:** Impact of BSA–morin nanoparticle incorporation on food matrices.

Property	Food Matrices					
	Whole Cow’s Milk		Reduced-Fat Milk		Skim Milk	
	Without NPs	2% Morin BSA NPS	Without NPs	2% Morin BSA NPS	Without NPs	2% Morin BSA NPS
Visual inspection	stable	stable	stable	stable	stable	presence of precipitate
Microscopy	uniform	uniform	uniform	uniform	uniform	uniform
Viscosity (20 °C)	2.0 mPa·s	2.4 mPa·s	1.7 mPa·s	1.9 mPa·s	1.5 mPa·s	1.7 mPa·s
pH	6.7	6.7	6.7	6.8	6.8	6.8
Color	white	faint creamy-yellow	white	faint creamy-yellow	white	faint creamy-yellow

## Data Availability

The manuscript contains the reported data. Additional relevant data can be obtained upon request from the corresponding authors.
